# Supplementary material for: Metabolic Profile of Patients with Smith-Magenis Syndrome: An Observational Study with Literature Review
Source: Children (Basel). 2023 Aug 25;10(9):1451. doi: 10.3390/children10091451 (PMC10527612; doi:10.3390/children10091451)
Supplement: Supplementary file 1 [file children-10-01451-s001.zip › children-2525839-supplementary.pdf]

Supplementary Table S1. Evaluation of the medical literature according to the Narrative Review Reporting Checklist used in our study.

	Item no.	Recommendation
**Title and abstract**	1	(*a*) Indicate the study's design with a commonly used term in the title or abstract
		(*b*) Provide in the abstract an informative and balanced summary of what was done and found
		**Introduction**
Background/rationale	2	Explain the scientific background and rationale for the investigation
Objectives	3	State specific objectives, including any hypotheses
		**Methods**
Study design	4	Present key elements of study design
Setting	5	Describe the setting, locations, and periods of recruitment, follow-up, and data collection
Participants	6	*Cohort and case-control study*—give the eligibility criteria, sources, and methods of selection of participants; give the rationale for the choice of cases and controls<br>*Cross-sectional study*—give the eligibility criteria, sources, and methods of selection of participants
		(*b*) *Cohort study*—for matched studies, give criteria and number of exposed and unexposed<br>*Case-control study*—for matched studies, give criteria and number of controls per case
Variables	7	Clearly define all outcomes, exposures, predictors, potential confounders, and effect modifiers
Data sources/measurement	8*	For each variable of interest, give sources of data and details of methods of assessment; describe comparability of assessment methods
Bias	9	Describe any efforts to address any sources of bias
Study size	10	Explain how the study size was defined
Quantitative variables	11	Explain how quantitative variables were handled in the analyses
Statistical methods	12	(*a*) Describe all statistical methods
		(*b*) Describe any methods used to examine subgroups
		(*c*) Explain how missing data were addressed
		(*d*) *Cohort study*—explain how loss to follow-up was addressed<br>*Case-control study*—explain how cases and controls were matched<br>*Cross-sectional study*—describe analytical methods
		(*e*) Describe any sensitivity analyses
		**Results**
Participants	13*	(a) Report numbers of individuals at each stage of study—e.g., potentially eligible, included in the study, completing follow-up, and analyzed
		(b) Give reasons for non-participation at each stage
		(c) Consider use of a flow diagram
Descriptive data	14*	(a) Give characteristics of study participants (e.g., demographic, clinical, social) and information on exposures to potential confounders
		(b) Indicate number of participants with missing data for each variable
		(c) *Cohort study*—summarize follow-up time
Outcome data	15*	*Cohort study*—report numbers of outcome events
		Case-control study—report numbers in each exposure category
		Cross-sectional study—report numbers of outcome events
Main results	16	(*a*) Give unadjusted estimates and confounder-adjusted estimates and their precision (e.g., 95% confidence interval)

		(*b*) Report category boundaries when continuous variables were categorized
		(*c*) Consider translating estimates of relative risk into absolute risk
Other analyses	17	Report other analyses done—e.g., analyses of subgroups and sensitivity analyses
	**Discussion**	
Key results	18	Summarize key results with reference to study objectives
Limitations	19	Discuss limitations of the study, taking into account sources of potential bias or imprecision
Interpretation	20	Give a cautious interpretation of results, considering objectives, limitations, multiplicity of analyses, and results from similar studies
Generalisability	21	Discuss the generalizability (external validity) of each result
	**Other information**	
Funding	22	Give the source of funding and role of the funders for the present study

*Information given separately for cases and controls in case-control studies and, if applicable, for exposed and unexposed groups in cohort and cross-sectional studies.

Note: Every article discusses each checklist item and gives methodological background and published examples of transparent reporting. The STROBE **(Strengthening the Reporting of Observational Studies in Epidemiology)** checklist is best used in conjunction with this article (freely available on the websites of PLoS Medicine at http://www.plosmedicine.org/, Annals of Internal Medicine at http://www.annals.org/, and Epidemiology at http://www.epidem.com/). Information on the STROBE Initiative is also available at www.strobe-statement.org.

Supplementary Table S2. Search strategy summary used in the medical literature evaluation of our study.

Items	Specification
Date of search	June 1st 2023
Databases and other sources searched	PubMed, Scopus, Cochrane Library
Combined search term used	“Smith-Magenis syndrome” AND “metabolic” OR “lipid metabolism”
Inclusion and exclusion criteria	Inclusion: (I) Case reports, case series, retrospective cohort series,<br>reviews, guidelines, consensus opinions; (II) English language; (III) Focusing on: Smith-Magenis syndrome, metabolic profile; (IV) Population: children and adults.<br>Exclusion: full-text unavailability
Initial results	N = 72
Final results	N = 16
